# PD-L1 expression as a predictor of postoperative recurrence and the association between the PD-L1 expression and *EGFR* mutations in NSCLC

**DOI:** 10.1038/s41598-021-96938-9

**Published:** 2021-09-01

**Authors:** Kensuke Kojima, Tetsuki Sakamoto, Takahiko Kasai, Tomoko Kagawa, Hyungeun Yoon, Shinji Atagi

**Affiliations:** 1 Department of General Thoracic Surgery, National Hospital Organization Kinki-Chuo Chest Medical Center, 1180 Nagasone-cho, Kita-ku, Sakai-shi, Osaka, 591-8555 Japan; 2 Department of Laboratory Medicine and Pathology, National Hospital Organization Kinki-Chuo Chest Medical Center, Osaka, Japan; 3 Department of Internal Medicine, National Hospital Organization Kinki-Chuo Chest Medical Center, Osaka, Japan; 4 Department of Thoracic Oncology, National Hospital Organization Kinki-Chuo Chest Medical Center, Osaka, Japan

**Keywords:** Medical research, Risk factors

## Abstract

Although information on the PD-L1 expression and *EGFR* mutations in non-small cell lung cancer (NSCLC) is important for therapeutic strategies, the effect of these factors on postoperative recurrence and the association between each factor have remained unclear. We retrospectively assessed the PD-L1 expression and *EGFR* mutations in 280 NSCLC patients, and analyzed the associations by multivariate analyses. The hazard ratio (HR) of postoperative recurrence in cases with high (≥ 50%) PD-L1 expression regarding negative expression was 4.83 (95% confidence interval [CI] 1.51–15.5). The HR for the PD-L1 expression, considered a continuous variable, was 1.016 (95% CI 1.01–1.03). The HRs in cases with *EGFR* major and minor mutations were 0.42 (95% CI 0.14–1.25) and 0.63 (95% CI 0.18–2.15), respectively. The high PD-L1 (≥ 50%) expression was significantly associated with exon 21 L858R mutation (Ex21) of *EGFR* (odds ratio, 0.10; 95% CI 0.01–0.87). The risk of postoperative recurrence increased 1.016-fold for every 1% increase in the PD-L1 expression, and a marked increase in risk was observed for expression levels of ≥ 50%. Whereas *EGFR* mutations were not an independent risk factor. The high PD-L1 (≥ 50%) expression was negatively associated with Ex21. These findings may help identify NSCLC patients with an increased risk of postoperative recurrence.

## Introduction

In recent years, the importance of immune checkpoint inhibitors (ICIs) in the treatment of lung cancer has increased^1^. Because the use of ICIs has been suggested to be potentially effective in the pre-surgical setting for surgically resectable non-small cell lung cancer (NSCLC), the benefits of ICIs in the surgical field have also been attracting attention^2^. The programmed death-ligand 1 (PD-L1) expression in tumors has already been shown to be a predictor of the treatment efficacy of ICIs for NSCLC^3^. On the other hand, epidermal growth factor receptor gene (*EGFR*) mutations are important in the pathogenesis of lung adenocarcinoma and EGFR tyrosine kinase inhibitor (EGFR TKI) treatment has been shown to significantly prolong survival of patients with lung adenocarcinoma with *EGFR* mutations^4^. Thus, the information on the PD-L1 expression and *EGFR* mutations in NSCLC are extremely important for therapeutic strategies.

The role of the PD-L1 expression and *EGFR* mutations in the postoperative prognosis of NSCLC and the association of the PD-L1 expression with *EGFR* mutations has been reported in several previous studies^5,6^. Recently, a large cohort study on postoperative patients with NSCLC suggested that the expression of PD-L1 might be a poor prognostic factor for recurrence-free survival (RFS)^7^. In addition, a large cohort study examining the association between the type of *EGFR* mutations and postoperative recurrence showed that patients with exon 19 deletion (Ex19) had significantly shorter postoperative RFS in comparison to those with exon 21 L858R mutation (Ex21)^8^. Regarding the association between the expression of PD-L1 and *EGFR* mutations in NSCLC, it has been reported that NSCLC with *EGFR* mutation was less likely to express PD-L1 and exhibited a poor response to ICIs in comparison to *EGFR* wild-type^9^.

However, the previous studies have not been considered simultaneously analyzing the interaction between different PD-L1 expression levels and various subtypes of *EGFR* mutation. Therefore, the details of the influence of these two factors on the postoperative prognosis and the understanding of the differential expression of PD-L1 depending on *EGFR* mutations remain unknown. By clarifying this association, it may be possible to identify NSCLC patients with a high risk of postoperative recurrence based on the information of the PD-L1 expression and *EGFR* mutations within the resected tumor. Furthermore, the knowledge on the association between PD-L1 expression and *EGFR* mutations may be useful in predicting the effect of ICIs on NSCLC patients with *EGFR* mutations.

The present study aimed to evaluate the effect of PD-L1 expression and *EGFR* mutations on postoperative recurrence in NSCLC, and their associations.

## Results

### Patient characteristics

The patient characteristics of each PD-L1 expression group are shown in Table 1. Chi-squared tests showed that the high expression of PD-L1 (tumor proportion score [TPS] of ≥ 50%) was more common in male patients, smokers, the histological type of SCC and other in NSCLC, pathological stage II and IIIA, the presence of adjuvant therapy, and wild-type *EGFR* mutation status. The PD-L1 expressing group tented to include a higher percentage of patients with *EGFR* mutation wild-type. The characteristics of patients categorized according to their *EGFR* mutation status are shown in Supplementary Table S1. The presence of *EGFR* mutations was associated with female sex, never smoker, ADC, earlier pathological stage, and low PD-L1 expression status of TPS of < 1% or 1–49%.

**Table 1:** Association between the PD-L1 expression and patient clinicopathological variables.

Variables	Total (N = 280)	PD-L1 expression			
		< 1% (N = 78)	1–49% (N = 146)	≥ 50% (N = 56)	*P*
Age: ≥ 70 years—no. (%)	156 (55.7)	39 (50.0)	88 (60.3)	29 (51.8)	0.27
Sex: male—no. (%)	157 (56.1)	30 (38.5)	84 (57.5)	43 (76.8)	< 0.001
Current or former smoker—no. (%)	174 (62.1)	40 (51.3)	87 (59.6)	47 (83.9)	< 0.001
**Histological type—no. (%)**					< 0.001
ADC	216 (77.1)	76 (97.4)	115 (78.7)	25 (44.6)	
SCC	46 (16.4)	1 (1.3)	22 (15.1)	23 (41.1)	
Other^a^	18 (6.4)	1 (1.3)	9 (6.2)	8 (14.3)	
**Pathological stage—no. (%)**					< 0.001
I	202 (72.1)	63 (80.8)	109 (74.7)	30 (53.6)	
II	50 (17.8)	12 (15.4)	23 (15.8)	15 (26.8)	
IIIA	28 (10.0)	3 (3.8)	14 (9.6)	11 (19.6)	
**Surgical procedure—no. (%)**					0.74
Lobectomy	262 (93.6)	76 (97.4)	135 (92.5)	51 (91.1)	
Bilobectomy	6 (2.1)	1 (1.3)	3 (2.1)	2 (3.6)	
Lobectomy with combined resection	12 (4.3)	1 (1.3)	8 (5.5)	3 (5.4)	
**Lymph node dissection—no. (%)**					< 0.001
ND1	25 (8.9)	17 (21.8)	7 (4.8)	1 (1.8)	
ND2	255 (91.1)	61 (78.2)	139 (95.2)	55 (98.2)	
**Adjuvant therapy—no. (%)**					0.018
Platinum-based chemotherapy	28 (10.0)	4 (5.1)	13 (8.9)	11 (19.6)	
***EGFR*mutation—no. (%)**					< 0.001
Wild-type	181 (64.6)	38 (48.7)	92 (63.0)	51 (91.1)	
Ex21	48 (17.1)	19 (24.4)	28 (19.2)	1 (1.8)	
Ex19	31 (11.1)	15 (19.2)	14 (9.6)	2 (3.6)	
Minor mutation^b^	20 (7.1)	6 (7.7)	12 (8.2)	2 (3.6)	

*PD-L1* programmed cell death-ligand 1, *ADC* adenocarcinoma, *SCC* squamous cell carcinoma, *EGFR* epidermal growth factor receptor gene, *Ex21* exon 21 L858R mutation, *Ex19* exon19 deletion mutation.

^a^Defined as histological types of NSCLC with the exclusion of ADC and SCC. Of 18 cases, 2 patients with large cell carcinoma, 3 patients with adenosquamous carcinoma, 7 patients with large cell neuroendocrine carcinoma and 6 patients with pleomorphic carcinoma.

^b^Defined as all mutations except Ex21 and Ex19.

### Recurrence-free survival

**Figure 1:**
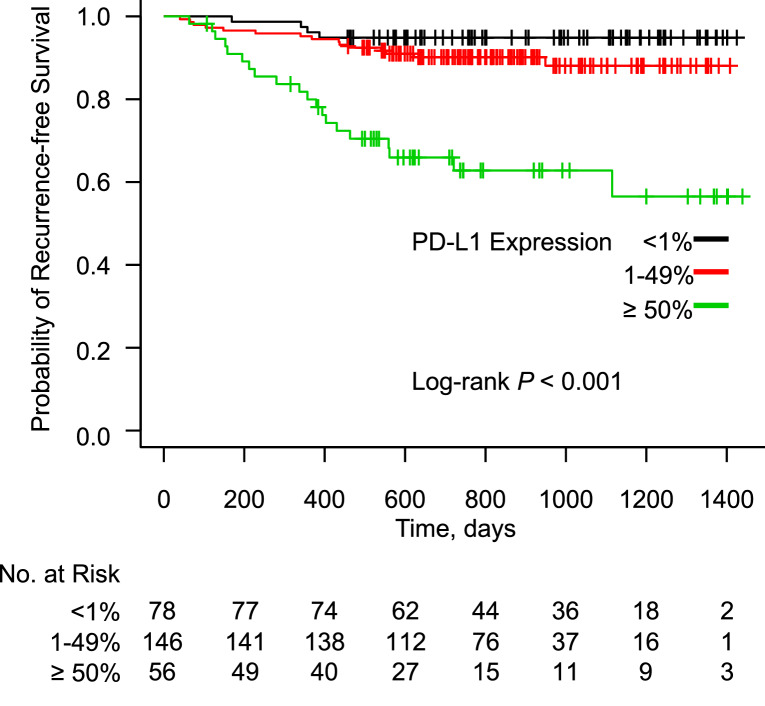
Kaplan–Meier curve showing the probability of a recurrence-free survival among patients after lung cancer resection according to the expression of PD-L1.

**Figure 2:**
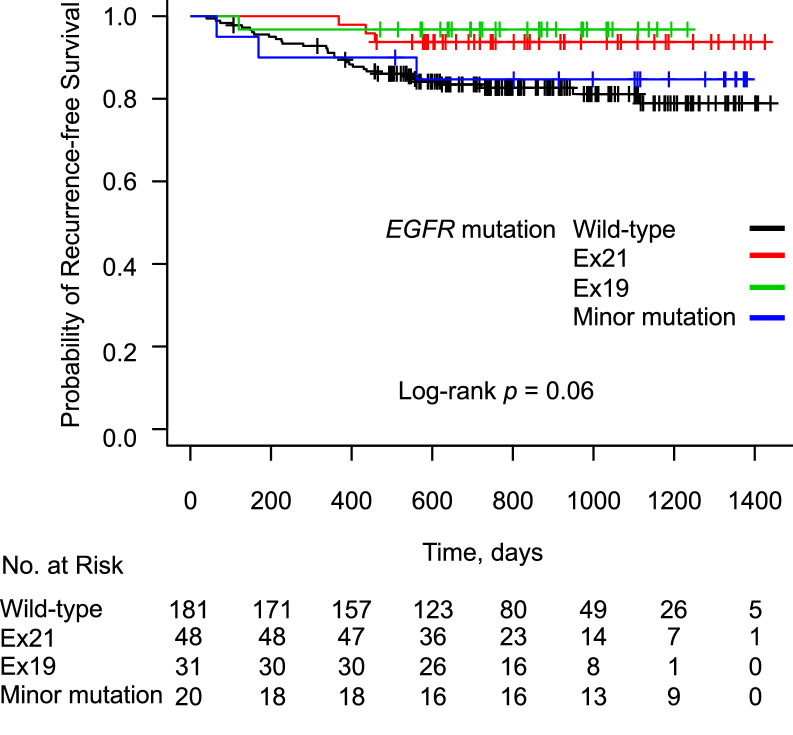
Kaplan–Meier curve showing the probability of a recurrence-free survival among patients after lung cancer resection according to the *EGFR* mutation status.

During a median follow-up period of 788 days after surgery, 39 patients developed recurrent disease (13.9%). The characteristics of patients with postoperative recurrence and those without recurrence are shown in Supplementary Table S2. Patients with postoperative recurrence showed a male predominance, and more commonly showed the following characteristics: smoker, histological type of SCC or other NSCLC, pathological stage II or IIIA, lobectomy with combined resection, the presence of adjuvant therapy, and their expression of PD-L1 was ≥ 50%. The Kaplan–Meier curves for RFS according to the PD-L1 expression status are shown in Fig. 1. RFS in the PD-L1-high group was significantly shorter in comparison to the PD-L1-negative and PD-L1-low groups, before adjustment for patient background (*P* < 0.001). The Kaplan–Meier curves for RFS according to the *EGFR* mutation status are shown in Fig. 2. Before adjustment for patient background, RFS did not differ among the *EGFR* mutation status groups (*P* = 0.06).

### The multivariate analyses of postoperative RFS according to the expression of PD-L1 and the *EGFR* mutation status

**Figure 3:**
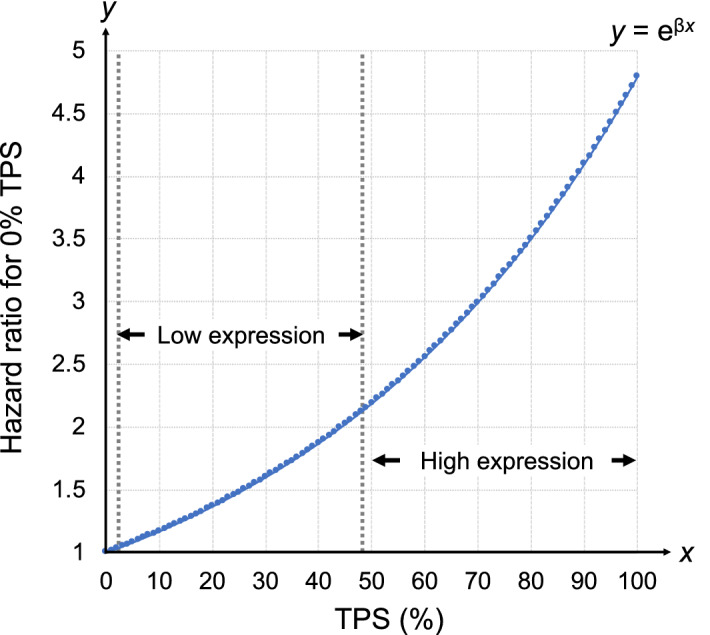
Hazard ratios of postoperative recurrence for each TPS value based on 0% TPS. The hazard ratio (*y*) for each TPS value was calculated based on the results of a multivariate Cox hazard proportional model with TPS (*x*)—considered as a continuous variable—as the explanatory variable. Since the regression coefficient (β) for this multivariate analysis was calculated to be 0.015662, the hazard ratio was given by the value of e^β^, i.e. 1.015786 ≈ 1.016. The function y was given by *y* = e^β*x*^.

The results of multivariate Cox proportional hazards analysis of factors associated with RFS are shown in Tables 2, 3 and 4. A significant differences in RFS was observed between the PD-L1-high group and the PD-L1-negative group (adjusted hazard ratio [HR], 4.83; 95% confidence interval [CI] 1.51–15.5) after adjustment for patient background factors including the *EGFR* mutation status (Table 2). RFS did not differ to a statistically significant extent in the PD-L1-low expression group (adjusted HR, 1.78; 95% CI 0.59–5.43) (Table 2). In the 39 recurrent cases, the numbers of patients in the PD-L1 non-expression, low expression, and high expression groups were 4, 15, and 20, respectively, suggesting a linear relationship between the PD-L1 expression and postoperative recurrence (Supplementary Table S2). The Cox proportional hazards analysis, in which PD-L1 expression level was considered a continuous variable rather than a categorical variable, showed that the expression of PD-L1 was significantly associated with postoperative recurrence, with or without adjustment for patient background factors (unadjusted HR, 1.025; 95% CI 1.02–1.03, adjusted HR, 1.016; 95% CI 1.01–1.03) (Table 3). Based on this adjusted HR, the results of plotting the HR toward each TPS value of the PD-L1 expression in the range of 0–100% are shown in Fig. 3. With a low TPS in the range of 1–49%, HRs ranged from 1.016 to 2.154. On the other hand, at a high expression level of 50–100%, HRs ranged from 2.188 to 4.788. In contrast, in the multivariate Cox proportional hazards analysis adjusted for patient background factors including the PD-L1 expression status, RFS did not differ to a statistically significant extent in the major mutation group (adjusted HR, 0.42; 95% CI 0.14–1.25) or the minor mutation group (adjusted HR, 0.63; 95% CI 0.18–2.15) with reference to the wild-type group of *EGFR* mutation (Table 4).

**Table 2:** The Cox proportional hazards analysis of RFS according to the PD-L1 expression.

PD-L1 expression	N	Recurrence N (%)	Unadjusted HR (95% CI), *P*	Adjusted HR^a^ (95% CI), *P*
< 1%	78	4 (5.13)	Reference	Reference
1–49%	146	15 (10.3)	2.12 (0.70–6.39), 0.18	1.78 (0.59–5.43), 0.31
≥ 50%	56	20 (35.7)	8.96 (3.01–26.3), < 0.001	4.83 (1.51–15.5), 0.008

*RFS* recurrence-free survival, *PD-L1* programmed death-ligand 1, *HR* hazard ratio, *CI* confidence interval, *EGFR* epidermal growth factor receptor gene.

^a^Adjusted by pathological stage, histological type, adjuvant chemotherapy and *EGFR* mutation status.

**Table 3:** Cox proportional hazards analysis of RFS according to the PD-L1 expression as a continuous variable.

	N	Recurrence N (%)	Unadjusted HR (95% CI), *P*	Adjusted HR^a^ (95% CI), *P*
PD-L1 expression^b^	280	39 (13.9)	1.025 (1.02–1.03), < 0.001	1.016 (1.01–1.03), 0.001

*RFS* recurrence-free survival, *PD-L1* programmed death-ligand 1, *HR* hazard ratio, *CI* confidence interval, *EGFR* epidermal growth factor receptor gene.

^a^Adjusted by pathological stage, histological type, adjuvant chemotherapy, *EGFR* mutation status and sex.

^b^The tumor proportion score (%) was as a continuous variable from 0 to 100.

**Table 4:** The Cox proportional hazards analysis of RFS according to the *EGFR* mutation status.

*EGFR* mutation	N	Recurrence N (%)	Unadjusted HR (95% CI), *P*	Adjusted HR^a^ (95% CI), *P*
Wild-type	181	32 (17.7)	Reference	Reference
Major mutation^b^	79	4 (5.06)	0.26 (0.09–0.74), 0.01	0.42 (0.14–1.25), 0.12
Minor mutation^c^	20	3 (15.0)	0.77 (0.23–2.51), 0.66	0.63 (0.18–2.15), 0.46

*RFS* recurrence-free survival, *EGFR* epidermal growth factor receptor gene, *HR* hazard ratio, *CI* confidence interval, *PD-L1* programmed death-ligand1, *Ex21* exon 21 L858R mutation, *Ex19* exon19 deletion mutation.

^a^Adjusted by PD-L1 expression status, pathological stage, histological type and adjuvant chemotherapy.

^b^Ex21 or Ex19.

^c^Defined as all mutations except Ex21 and Ex19.

### The associations between the expression of PD-L1 and *EGFR* mutations

A multinomial logistic regression analysis was performed to examine the link between the expression of PD-L1 and *EGFR* mutations (Table 5). None of the *EGFR* mutations were significantly associated with the low expression of PD-L1: Ex21 (adjusted OR, 0.87; 95% CI 0.41–1.85); Ex19 (adjusted OR, 0.54; 95% CI 0.23–1.30); minor mutation (adjusted OR 1.05; 95% CI 0.35–3.22). The high expression of PD-L1 showed a significant negative association with Ex21 mutation of *EGFR* (adjusted OR, 0.10; 95% CI 0.01–0.87). Neither Ex19 nor minor *EGFR* mutations showed a significant association with the high expression of PD-L1: Ex19 (adjusted OR, 0.23; 95% CI 0.04–1.22); minor mutation (adjusted OR, 0.38; 95% CI 0.06–2.50).

**Table 5:** The multinomial logistic regression analysis to investigate the association between the expression of PD-L1 and *EGFR* mutations.

*EGFR* mutation	PD-L1 expression			
	1–49% versus < 1%		≥ 50% versus < 1%	
	Unadjusted OR (95% CI), *P*	Adjusted OR^a^ (95% CI)**,** *P*	Unadjusted OR (95% CI)**,** *P*	Adjusted OR^a^ (95% CI)**,** *P*
Wild-type	Reference	Reference	Reference	Reference
Ex21	0.61 (0.30–1.22), 0.16	0.87 (0.41–1.85), 0.72	0.04 (0.01–0.31), < 0.001	0.10 (0.01–0.87), 0.03
Ex19	0.39 (0.17–0.88), 0.02	0.54 (0.23–1.30), 0.17	0.09 (0.02–0.46), < 0.001	0.23 (0.04–1.22), 0.08
Minor mutation^b^	0.83 (0.29–2.36), 0.72	1.05 (0.35–3.22), 0.93	0.25 (0.04–1.30), 0.09	0.38 (0.06–2.50), 0.31

*PD-L1* programmed death-ligand 1, *EGFR* epidermal growth factor receptor gene, *OR* odds ratio, *CI* confidence interval, *Ex21* exon 21 L858R mutation, *Ex19* exon19 deletion mutation.

^a^Adjusted by age, sex, smoking status, pathological stage and histological type.

^b^Defined as all mutations except Ex21 and Ex19.

## Discussion

The multivariate Cox proportional hazards analysis adjusted for *EGFR* mutation status, pathological stage, histological type, and adjuvant chemotherapy revealed that postoperative recurrence of lung cancer was 4.8 times as likely to occur with a PD-L1-high expression status in comparison to PD-L1-negative cases. In addition, the Cox proportional hazards analysis, in which the PD-L1 expression was considered as a continuous variable, showed that a 1% elevation of the TPS in the PD-L1 expression increased the risk for postoperative recurrence 1.016-fold. The Cox proportional hazards analysis with adjustment for the expression of PD-L1, pathological stage, histological type and adjuvant chemotherapy revealed that *EGFR* mutations were not significantly associated with postoperative recurrence. A multinomial logistic regression analysis showed a significant negative association between the high expression of PD-L1 and the presence of the Ex21 mutation.

Previous studies have not shown consistent results regarding the prognosis associated with the expression of PD-L1 and *EGFR* mutations in NSCLC. One study showed that the low expression of PD-L1 was associated with significantly reduced RFS in NSCLC patients with *EGFR* mutations^10^. However, this study did not show a significant association between the high expression of PD-L1 and RFS. In addition, this study focused solely on the influence of the presence or absence of *EGFR* mutations, and the interaction between the PD-L1 expression and various *EGFR* mutations was not considered in the analysis. Another study reported that the expression of PD-L1 is a favorable prognostic factor for postoperative overall survival (OS) in NSCLC^6^. However, in that study, only factors that were identified as significant in a univariate analysis were selected as explanatory variables for the multivariate analysis, and the effects of important confounding factors that may affect OS, such as histological type and the presence of adjuvant chemotherapy, were not taken into a consideration. It should also be noted that PD-L1 expression was categorized into < 50% and ≥ 50% as a comparison group. These elements may have had a significant impact on the trend, which was opposite the trend observed in our study. Furthermore, another study showed that the patients with Ex21 had a significantly longer RFS in comparison to those with WT or Ex19^8^. However, this study did not evaluate RFS, with the inclusion of the PD-L1 expression as a confounding factor in the multivariate analysis.

In the present study, we performed multivariate analyzes considering the interaction of different PD-L1 expression levels with *EGFR* mutations in NSCLC and revealed the following three novel points. First, patients with the high expression of PD-L1 had significantly shorter postoperative RFS, whereas those with the low expression of PD-L1 showed no significant difference. In previous studies, the results were not consistent because the cutoff value of PD-L1 was set for each study or the explanatory variables to be included in the multivariate analysis were determined based on the results of a univariate analysis. In contrast, we ensured reproducibility by adopting the classification of the PD-L1 expression, which is frequently used in general clinical practice, and by determining the explanatory variables in advance. As a result, we clearly showed that expression levels of ≥ 50% was significantly correlated with postoperative RFS, while expression levels of 1–49% were not significantly correlated. On the other hand, in the rough classification of the PD-L1 expression used in clinical practice, for example, expression levels of 1–49% and 50–100% were treated as the same respectively; thus, the effect of each 1% increase in the expression of PD-L1 on postoperative recurrence has not been studied. In this study, we quantitatively evaluated the risk of recurrence for each 1% increase in the PD-L1 expression using the PD-L1 expression, which was regarded as a continuous variable, as an explanatory variable in the multivariate Cox hazard analysis. We found that, with expression levels in the range of 1–49% expression, the risk varied by a factor of 1.5 when the expression was ≤ 30% and by a factor of 2 when it was 40%, but when the expression was above 50%, the risk tended to exceed twofold, reaching 4.8-fold at 100% expression. In other words, our study showed that the risk varies even within the low expression group (1–49%) and that it may vary even more within the high expression group (≥ 50%). To the best of our knowledge, this is the first report to conduct such an evaluation of the PD-L1 expression in NSCLC. Second, the presence of *EGFR* mutations did not contribute significantly to postoperative RFS. This report may be the first to show that *EGFR* mutations are not associated with postoperative RFS, regardless of the type of *EGFR* mutation, considering the interaction with the PD-L1 expression patterns in the multivariate analysis. Third, the high expression of PD-L1 showed a significant negative connection with Ex21. Although there have been many reports of negative association between *EGFR* mutations and PD-L1, most of them identified these associations by univariate analyses, and few studies have reported the association in multivariate analysis with the removal of confounding factors. The novelty of our study is that we showed—based on a multivariate analysis—that the association between the PD-L1 expression and *EGFR* mutation differs depending on the type of *EGFR* mutation.

These three points may be biologically plausible. First, the high expression of PD-L1 in NSCLC may affect the postoperative prognosis. Because cancer cells inactivate T cells via the PD-L1 expression^11^, the higher the expression of PD-L1, the more the immune system is suppressed, which may lead to greater cancer progression than expected after surgery. Some studies have reported that the expression of PD-L1 was associated with the AKT-mTOR pathway, which has been shown to be necessary for cell proliferation^12^. Therefore, the expression of PD-L1 may be correlated with oncogenic signal, the high expression of which may be involved in high tumor progression. Second, *EGFR* mutations in NSCLC may not affect postoperative recurrence. An in vitro study using cell lines have reported that *EGFR* mutations promote the expression of PD-L1^13^. However, in vivo, it has been reported that various cytokines in the tumor microenvironment affect the PD-L1 expression on cancer cells^14^. An experiment in which cell lines were stimulated with cytokines, such as interferon-γ due to mimicking of the in vivo environment demonstrated that the expression of PD-L1 is higher in cells with wild-type *EGFR* in comparison to those with *EGFR* mutations^15^. In the in vivo tumor microenvironment, *EGFR* mutation may not be an independent risk factor for recurrence because of attenuated immunosuppression in connection with the tendency for low PD-L1 expression levels in cells with *EGFR* mutations. Third, among the *EGFR* mutation subtypes, Ex21 may be negatively associated with the high expression of PD-L1 in NSCLC. The tumor mutation burden (TMB) has been known to be an effective predictor of an improved response to ICIs in NSCLC^16^. One study suggested that there was no relevant association between the expression of PD-L1 and a high TMB in tumors^17^. In addition, another study examining the association between *EGFR* mutations and the TMB reported that the TMB tended to be higher in cells with Ex21 than in those with Ex19^18^. Thus, the high TMB in cancer cells with Ex21 may contribute to the suppression of the high expression of PD-L1.

The present study was associated with some limitations. First, because the number of variables that could be included in the multivariable Cox proportional hazards analysis was limited due to the small number of postoperative recurrence cases, it was not possible to incorporate all of other confounding factors into the model at once. A larger sample size using a model that simultaneously includes all variables is needed in order to evaluate the influence of the PD-L1 expression and *EGFR* mutation status on the postoperative recurrence with higher accuracy. In addition, in the multinomial logistic regression analysis in our cohort the sample size of the high PD-L1 expression group was very small (Ex21, n = 1; Ex19, n = 2). In general, small sample sizes have low power, which increases the probability of type 2 errors. However, in our multivariate analysis, the relationship between the high PD-L1 expression and Ex21 was statistically significant, so the problem of type 2 error was overcome. The possibility of a type 1 error was also reduced by the significance level. Therefore, the relationship between the high expression of PD-L1 and Ex21, which showed statistical significance, even with our sample size, may be robust. On the other hand, Ex19 did not show a significant difference in our multivariate analysis, and we cannot deny the possibility that a type 2 error may have occurred due to the small sample size. It is known that the larger the sample size, the higher the power of detection. Therefore, the association between the high expression of PD-L1 and Ex19 should be evaluated and confirmed with a larger sample. Second, our study was limited to surgical cases; we did not examine advanced cases that were managed without surgery. *EGFR* mutation positivity was reportedly associated with the expression of PD-L1 in stage IV^19^. This result is completely opposite to the results of our study. Therefore, the PD-L1 expression in advanced stage may behave differently than other stages. A prognostic analysis that analyzed the influence of the PD-L1 expression and *EGFR* mutation status, including patients with advanced stage disease, may also be needed.

In summary, we performed multivariate analyses using a cohort of patients who had undergone complete surgical resection for lung cancer to compare postoperative RFS between groups categorized according to their PD-L1 expression and *EGFR* mutation status. We showed that ≥ 50% PD-L1 positivity within the resected tumor tissue was an independent risk factor for recurrence after lung cancer surgery, whereas the *EGFR* mutation status was not. We quantitatively assessed the increased risk for each 1% of the PD-L1 expression. We then showed that the risk also varies widely within each of the clinically used classifications of the PD-L1 expression. In addition, we showed that Ex21 may be not a risk factor for postoperative recurrence due to the difficulty in expressing a high level of PD-L1. Our findings may contribute to the selection of patients who are eligible for adjuvant chemotherapy using ICIs, regardless of pathological stage.

## Methods

### Patients

A total of 280 patients with NSCLC who had undergone surgical lung resection at Kinki-Chuo Chest Medical Center (KCMC) from April 2017 to January 2020 were included in our study. We selected the patients who had undergone complete resection. Those who had received limited resection were excluded because a previous report showed that limited resection was associated with a higher rate of locoregional recurrence in comparison to lobectomy^20^. The histopathological diagnosis according to the current 2015 World Health Organization classification was performed by pathologists^21^. Adjuvant chemotherapy with a platinum-based regimen, according to the guidelines of the Japanese Lung Cancer Association, was administered to patients who were eligible and who had given their informed consent. Patients who received neoadjuvant therapy were excluded because it was reported that the PD-L1 expression status of cancer cells was altered after neoadjuvant chemotherapy^22^. Patients who had participated in postoperative clinical trials of adjuvant TKIs or adjuvant immunotherapy in which KCMC had been participating were also excluded because of the difficulty in assessing the efficacy of the study drugs considering the possibility of placebo administration. Other clinicopathological features, including age, sex, smoking status, pathologic tumor-node-metastasis (TNM) classification (eighth edition) and the presence of postoperative recurrence were collected from medical records. The present study was approved by the Institutional Review Board of KCMC (Approval number: 725). Informed consent was obtained by an opt-out method using the website of our institution. All methods were performed in accordance with relevant guidelines and regulations.

### Tumor PD-L1 immunohistochemistry and the *EGFR* mutation assay

All viable cancer cells on the entire pathological tissue section of each tumor sample were evaluated. We used the PD-L1 clone 22C3 pharmDx kit and Dako Automated Link 48 platform (Agilent Technologies, Dako, Carpinteria, CA, USA) to measure the PD-L1 expression. The PD-L1 tumor proportion score (TPS) was calculated as the percentage of complete or partial membrane staining in a sample. The cut-off value for the expression of PD-L1 was set at 50% and 1% based on a previous clinical trial^23^. The tumor samples of each patient were separated into 3 groups based on the presence of positivity stained cells in specimen, as follows: < 1% (negative), 1–49% (low expression), and ≥ 50% (high expression). All patients were subjected to an *EGFR* mutation assay by the testing laboratories (Cobas EGFR Mutation Test; Roche Molecular Diagnostics, Pleasanton, CA, USA).

### Recurrence-free survival

The primary outcome of this study was recurrence-free survival (RFS), defined as the time from the date of curative resection to the date on which disease relapse was diagnosed. The patients received blood tests and a chest X-ray after surgery every 3 or 6 months. Additional screenings were performed in cases where abnormal findings were observed. The diagnosis of relapse was comprehensively determined based on the results of examinations.

### Statistical analyses

Chi-squared tests were used to compare the proportions of categorical variables between each of the PD-L1 expression groups and *EGFR* mutation groups. The probability of RFS was assessed using the Kaplan–Meier method and log-rank tests. A multivariate Cox proportional hazards analysis was performed to estimate the hazard ratios (HRs) with adjustment by risk factors for recurrence. A multinomial logistic regression analysis was performed to assess the odds ratios (ORs) for each *EGFR* mutation status in the PD-L1-high and PD-L1-low groups, with the PD-L1-negative group as a reference. A multivariate Cox proportional hazards analysis and a multivariate logistic regression analysis can analyze the covariates of the number of cases with an outcome divided by 10 or 5^24,25^. In this study, the number in the Cox proportional hazards analysis was 39 (i.e., the number of cases with relapse) divided by 10 or 5 (result: 4 or 8). And the number of cases included in the logistic regression analysis was 56 (i.e., the number of cases with the high expression of PD-L1) divided by 10 or 5 (coming to 5 or 11). We selected the following 8 factors in a multivariate Cox hazards analysis: the PD-L1 expression level (low and high [reference: negative]); histological type (squamous cell carcinoma (SCC) and other types [reference: adenocarcinoma (ADC)]); pathological stages (stage II–IIIA [reference: stage I]); adjuvant chemotherapy (platinum [reference: none]); the *EGFR* mutation status (major mutation (Ex19 or Ex21) and minor mutation (all mutations except Ex21 and Ex19) [reference: wild-type]). We also conducted an evaluation where the PD-L1 expression level was considered as a continuous variable rather than a categorical variable. In this case, since the PD-L1 expression level was counted as one factor, a total of eight factors were included in the multivariate analysis, including sex as a new factor in addition to the above factors. These factors have been reported to evaluate factors associated with postoperative recurrence^26^. In addition, we selected the following 10 factors in the multinomial logistic regression analysis: histological types (SCC and other types [reference: ADC]); pathological stages (stage II and stage IIIA [reference: stage I]); the *EGFR* mutation status (Ex21, Ex19 and minor mutation [reference: wild-type]); age; sex; and smoking status. All statistical analyses were conducted using EZR (Saitama Medical Center, Jichi Medical University, Saitama, Japan), which is a graphical user interface for R (The R Foundation for Statistical Computing, Vienna, Austria). EZR is a modified version of R commander with added biostatistical functions^27^. *P* values of < 0.05 were considered to indicate statistical significance.

## Supplementary Information

Supplementary Table S1.

Supplementary Table S2.

## Abbreviations

ADC: Adenocarcinoma
*EGFR*: Epidermal growth factor receptor gene
Ex19: Exon 19 deletion
Ex21: Exon 21 L858R
NSCLC: Non-small cell lung cancer
PD-L1: Programmed death-ligand 1
RFS: Recurrence free survival
SCC: Squamous cell carcinoma
