# Supplementary material for: PD-L1 expression as a predictor of postoperative recurrence and the association between the PD-L1 expression and EGFR mutations in NSCLC
Source: Sci Rep. 2021 Sep 1;11:17522. doi: 10.1038/s41598-021-96938-9 (PMC8410871; doi:10.1038/s41598-021-96938-9)
Supplement: Supplementary file 1 — Supplementary Table S1. [file 41598_2021_96938_MOESM1_ESM.pdf]

Title.

PD-L1 expression as a predictor of postoperative recurrence and the association between the PD-L1 expression and *EGFR* mutations in NSCLC

Authors' names:

Kensuke Kojima, Tetsuki Sakamoto, Takahiko Kasai, Tomoko Kagawa, Hyungeun Yoon & Shinji Atagi

Supplementary Table S1. Association between the *EGFR* mutation status and patient clinicopathological variables

Variables	Total (N = 280)	***EGFR* mutation status** Wild-type (N = 181)	Ex21 (N = 48)	Ex19 (N = 31)	Minor mutation [a] (N = 20)	*P*
Age: ≥70 years — no. (%)	156 (55.7)	97 (53.6)	33 (68.8)	17 (54.8)	9 (45.0)	0.21
Sex: Male — no. (%)	157 (56.1)	123 (68.0)	16 (33.3)	12 (38.7)	6 (30.0)	< 0.001
Current or former smoker — no. (%)	174 (62.1)	135 (74.6)	19 (39.6)	13 (41.9)	7 (35.0)	< 0.001
Histological Type — no. (%)						<0.001
ADC	216 (77.1)	120 (66.3)	48 (100)	30 (96.8)	18 (90.0)	
SCC	46 (16.4)	46 (25.4)	0 (0)	0 (0)	0 (0)	
Other [b]	18 (6.4)	15 (8.3)	0 (0)	1 (3.2)	2 (10.0)	
Pathological Stage — no. (%)						0.02
I	202 (72.1)	128 (70.7)	40 (83.3)	20 (64.5)	14 (70.0)	
II	50 (17.9)	37 (20.4)	3 (6.2)	9 (29.0)	1 (5.0)	
IIIA	28 (10.0)	16 (8.8)	5 (10.4)	2 (6.5)	5 (25.0)	
Surgical procedure — no. (%)						0.74

Lobectomy	262 (93.6)	167 (92.4)	46 (95.8)	31 (100)	18 (90.0)	
Bilobectomy	6 (2.1)	6 (3.3)	0 (0)	0 (0)	0 (0)	
Lobectomy with combined resection	12 (4.3)	8 (1.3)	2 (4.2)	0 (0)	2 (10.0)	
Lymph node dissection — no. (%)						0.97
ND1	25 (8.9)	15 (8.3)	5 (10.4)	3 (9.7)	2 (10.0)	
ND2	255 (91.1)	166 (91.7)	43 (89.6)	28 (90.3)	18 (90.0)	
Adjuvant therapy — no. (%)						0.72
Platinum-based chemotherapy	28 (10.0)	19 (10.5)	3 (6.2)	3 (9.7)	3 (15.0)	
PD-L1 expression — no. (%)						<0.001
<1%	78 (27.9)	38 (21.0)	19 (39.6)	15 (48.4)	6 (30.0)	
1-49%	146 (52.1)	92 (50.8)	28 (58.3)	14 (45.2)	12 (60.0)	
≥50%	56 (20.0)	51 (28.2)	1 (2.1)	2 (6.5)	2 (10.0)	

[a] Defined as all mutations except Ex21 and Ex19.

[b] Defined as histological types of NSCLC with the exclusion of ADC and SCC. The 18 cases included 2 patients with large cell carcinoma, 3 patients with adenosquamous carcinoma, 7 patients with large cell neuroendocrine carcinoma and 6 patients with pleomorphic carcinoma.

EGFR epidermal growth factor receptor gene, *Ex21* exon 21 L858R mutation, *Ex19* exon19 deletion mutation, *ADC*

adenocarcinoma, *SCC* squamous cell carcinoma, *PD-L1* programmed cell death-ligand 1.
