# Supplementary material for: PD-L1 expression as a predictor of postoperative recurrence and the association between the PD-L1 expression and EGFR mutations in NSCLC
Source: Sci Rep. 2021 Sep 1;11:17522. doi: 10.1038/s41598-021-96938-9 (PMC8410871; doi:10.1038/s41598-021-96938-9)
Supplement: Supplementary file 2 — Supplementary Table S2. [file 41598_2021_96938_MOESM2_ESM.pdf]

Title.

PD-L1 expression as a predictor of postoperative recurrence and the association between the PD-L1 expression and *EGFR* mutations in NSCLC

Authors' names:

Kensuke Kojima, Tetsuki Sakamoto, Takahiko Kasai, Tomoko Kagawa, Hyungeun Yoon & Shinji Atagi

Supplementary Table S2. Association between postoperative recurrence and patient clinicopathological variables.

variables	Total (N = 280)	No recurrence (N =241)	Recurrence (N = 39)	*P*
Age: ≥70 years — no. (%)	156 (55.7)	135 (56.0)	21 (53.8)	0.86
Sex: Male — no. (%)	157 (56.1)	127 (52.7)	30 (76.9)	0.005
Current or former smoker — no. (%)	174 (62.1)	143 (59.3)	31 (79.5)	0.02
Histological Type — no. (%)				0.006
ADC	216 (77.1)	192 (79.7)	24 (61.5)	
SCC	46 (16.4)	38 (15.8)	8 (20.5)	
Other [a]	18 (6.4)	11 (4.6)	7 (17.9)	
Pathological Stage — no. (%)				<0.001
I	202 (72.1)	189 (78.4)	13 (33.3)	
II	50 (17.8)	40 (16.6)	10 (25.6)	
IIIA	28 (10.0)	12 (5.0)	16 (41.0)	
Surgical procedure — no. (%)				0.011

Lobectomy	262 (93.6)	229 (95.0)	33 (84.6)	
Bilobectomy	6 (2.1)	5 (2.1)	1 (2.6)	
Lobectomy with combined resection	12 (4.3)	7 (2.9)	5 (12.8)	
Lymph node dissection — no. (%)				0.22
ND1	25 (8.9)	24 (10.0)	1 (2.6)	
ND2	255 (91.1)	217 (90.0)	38 (97.4)	
Adjuvant therapy — no. (%)				<0.001
Platinum-based chemotherapy	28 (10.0)	15 (6.2)	13 (33.3)	
EGFR mutation — no. (%)				0.051
Wild-type	181 (64.6)	149 (61.8)	32 (82.1)	
Ex21	48 (17.1)	45 (18.7)	3 (7.7)	
Ex19	31 (11.1)	30 (12.4)	1 (2.6)	
Minor mutation [b]	20 (7.1)	17 (7.1)	3 (7.7)	
PD-L1 expression				<0.001
<1%	78 (27.8)	74 (30.7)	4 (10.3)	
1-49%	146 (52.1)	131 (54.4)	15 (38.5)	

≥50%	56 (20.0)	36 (14.9)	20 (51.3)

[a] Defined as histological types of NSCLC with the exclusion of ADC and SCC. The 18 cases included 2 patients with large cell carcinoma, 3 patients with adenosquamous carcinoma, 7 patients with large cell neuroendocrine carcinoma and 6 patients with pleomorphic carcinoma.

[b] Defined as all mutations except Ex21 and Ex19.

PD-L1 programmed cell death-ligand 1, *ADC* adenocarcinoma, *SCC* squamous cell carcinoma, *EGFR* epidermal growth factor receptor gene, *Ex21* exon 21 L858R mutation, *Ex19* exon19 deletion mutation.
